# Effects of *Chlorella pyrenoidosa* Amendment on Tetracycline–Copper Dissipation, Soil Microbial Communities, and Tetracycline Resistance Genes

**DOI:** 10.3390/microorganisms14092065

**Published:** 2026-09-16

**Authors:** Yang Wu, Yang Feng, Bo Wu, Yu Gao, Jingwen Xu

**Affiliations:** 1 School of Environmental and Chemical Engineering, Shenyang University of Technology, Shenyang 110870, China; 2 Technical Centre for Soil, Agriculture and Rural Ecology and Environment, Ministry of Ecology and Environment, Beijing 100012, China; 3 Key Laboratory of Karst Georesources and Environment, Ministry of Education, College of Resources and Environmental Engineering, Guizhou University, Guiyang 550025, China; 4 Institute of Applied Ecology, Chinese Academy of Sciences, Shenyang 110016, China

**Keywords:** *Chlorella*, antibiotic resistance genes, copper immobilization, antibiotic dissipation, soil microbiome

## Abstract

Tetracycline (TC)–copper (Cu) co-contamination can impair soil functioning and intensify selection for antibiotic resistance. In a 49-day microcosm experiment, we evaluated the effects of *Chlorella pyrenoidosa* supplementation on soils treated with TC (0, 30, or 100 mg·kg^−1^) and Cu (0, 100, or 500 mg·kg^−1^). TC removal, extractable Cu, soil physicochemical properties, enzyme activities, tetracycline resistance genes (TRGs), and microbial community composition were assessed. In unamended contaminated microcosms, TC dissipation ranged from 22.7% to 43.5%, whereas *C. pyrenoidosa* amendment increased TC dissipation to 49.9–73.5%. The addition also reduced extractable Cu by 35.6–50.6%, partially restored dehydrogenase and catalase activities, and altered bacterial community structure. Metagenomic analysis showed that *tetX* was the predominant TRG detected and that algal supplementation was associated with lower total TRG signals and reduced relative abundances of several pollution-associated taxa, including *Rhodanobacter*. Correlation analyses revealed associations among TRGs, microbial taxa, and treatment conditions but did not establish direct host–gene relationships or horizontal gene transfer. Overall, *C. pyrenoidosa* application enhanced TC removal and Cu immobilization and was associated with reduced enrichment of the tetracycline resistome.

## 1. Introduction

The widespread co-occurrence of antibiotics and heavy metals in agricultural soils has emerged as a pressing environmental concern, posing potential risks to ecosystem functioning and human health through the spread of antimicrobial resistance. These contaminants not only persist in soil but also interact with each other through complexation, with their mobility and toxicity critically modulated by soil properties such as pH, organic matter, and ionic strength [1]. High-throughput sequencing analysis of agricultural soils by Jing et al. revealed that co-contamination reduced soil dehydrogenase activity [2]. Wang et al. confirmed that SOM is a core driver mediating the expression of soil enzyme activities, with its regulatory efficiency directly correlated with the degree of tetracycline TC–Cu co-contamination in soil [3]. These findings indicate that the combined residues of tetracycline and Cu in livestock and poultry manure can complex with SOM, thereby enriching antibiotic-resistant microorganisms [4]. Meanwhile, the extensive use of antibiotics has rendered antibiotic resistance genes (ARGs) a new class of environmental pollutants, which persist in the environment long-term due to the uncertainty of gene transfer and the emergence of a large number of antibiotic-resistant bacteria [5]. Knapp et al. investigated the relationship between heavy metals and ARGs in soil, demonstrating positive correlations between the levels of various ARGs and Cu [6]. Additionally, De la Iglesia et al. found that the concentrations of antibiotics and heavy metals were significantly correlated with ARG abundances, and the coexistence of these two types of pollutants might further increase ARG abundances [7]. Thus, it is hypothesized that heavy metal-antibiotic co-contamination exerts a synergistic selection effect.

Among various remediation technologies, physical remediation, chemical-material synergistic remediation, microbial-enhanced remediation, and multi-technology combination approaches are key strategies for the efficient mitigation of co-contamination. Zheng et al. [8] used composite materials to remediate the combined pollution of Cu^2+^ and tetracycline (TC) in soil, and the results showed that the maximum adsorption capacities of the material for Cu^2+^ and TC reached 128.3 mg/g and 8 mg/g, respectively. Wang et al. [9] achieved a TC degradation rate of 99.1% within 168 h in the presence of 5 mmol/L heavy metal ions, and it also increased the removal efficiencies of Cr and Co by 62.9% and 80.0%, with a significant reduction in the toxicity of degradation products. In the field of microbial remediation, *Chlorella* exhibits remarkable advantages in treating multiple types of pollution (e.g., heavy metals, antibiotics, and industrial wastewater) due to its rapid growth rate, strong environmental adaptability, and high resource recovery potential. Among various microalgae, *Chlorella pyrenoidosa* is a unicellular green alga with rapid growth, strong environmental adaptability, and abundant surface functional groups (e.g., hydroxyl, carboxyl) that facilitate both heavy metal adsorption and organic pollutant degradation. It was selected here for its proven tolerance to combined TC–Cu stress and its well-documented dual removal mechanisms via extracellular complexation and intracellular biotransformation. Ji et al. [10] demonstrated the significant synergistic removal effect of *Chlorella* on sulfadiazine (SDZ) and Cu. Wu et al. [11] developed an immobilized *Alcaligenes faecalis-Chlorella* combined system, which achieved a TC removal rate of up to 99.0% in heavy metal-containing medical and aquaculture wastewater. The remediation mechanism of *Chlorella* involves the synergistic enrichment of heavy metals through complexation and adsorption by extracellular polymeric substance (EPS) functional groups and intracellular accumulation, as well as the degradation of tetracycline via metabolic transformation [12], thereby reducing the contents of tetracycline and heavy metals. However, current research on *Chlorella* in the mitigation of soil co-contamination by heavy metals and antibiotics remains scarce. Existing studies have mostly focused on aquatic environments or the remediation of single pollutants, with a clear lack of systematic research on the complex soil matrix. Previous studies have confirmed that Chlorella can reduce the bioavailability of soil heavy metals through extracellular adsorption and speciation transformation [13], and it can synergistically remove antibiotics and heavy metals in water. There is limited research on the actual removal efficiencies of Chlorella for antibiotics (e.g., tetracyclines, sulfonamides) and the mitigation efficiencies for heavy metals (e.g., Cu) under conditions of soil co-contamination. Furthermore, soil co-contamination tends to affect ARG abundances by altering pollutant migration and transformation characteristics, yet the regulatory effects, potential mechanisms, and long-term environmental risks of *Chlorella* remediation on soil TRGs (e.g., sul and tet genes) have not been thoroughly investigated [14].

Based on the actual TC-Cu pollution background of agricultural soils in northern China and the research gaps mentioned above, this study took soils contaminated by single and combined TC-Cu as the research objects, aiming to: (1) systematically investigate the regulatory effects of *Chlorella pyrenoidosa* on soil physicochemical properties, enzyme activities, and pollutant degradation efficiencies, and reveal the interaction mechanism between *Chlorella pyrenoidosa* and co-contaminated soils; (2) further strengthen the research on the remediation efficiency of *Chlorella pyrenoidosa* under soil co-contamination scenarios, and clarify the synergistic mitigation laws of antibiotics and heavy metals; (3) systematically analyze the impacts of the remediation process on the distribution and diffusion of soil TRGs, and reveal the TRG regulation mechanism of *Chlorella pyrenoidosa* from the perspective of microbial community-host gene interaction. This study is expected to provide a theoretical basis and technical support for the practical application of *Chlorella pyrenoidosa* in the remediation of heavy metal-antibiotic co-contaminated agricultural soil.

## 2. Materials and Methods

### 2.1. Algae, Soil, and Reagents

Agricultural soil was collected from the 5–10 cm layer of a field in the Shenyang Economic and Technological Development Zone, Liaoning, China. Roots and plant residues were removed, and the soil was air-dried, gently ground, and passed through a 10 mm stainless-steel sieve. Initial properties were pH 6.52, EC 1040 µS·cm^−1^, SOM 2015 mg·kg^−1^, and total Cu 22.3 mg·kg^−1^; TC was not detected.

TC (purity 98%) was obtained from Shanghai Aladdin Biochemical Technology Co., Ltd. (Shanghai, China). Copper sulfate pentahydrate (CuSO_4_·5H_2_O, analytical grade) was obtained from Sinopharm Chemical Reagent Co., Ltd. (Shanghai, China). *C. pyrenoidosa* biomass (1 g dry weight·mL^−1^) was purchased from Shenyang Kemio Chemical Trade Co., Ltd. (Shenyang, China) and stored prior to soil application. Experimental loadings were TC at 30 or 100 mg·kg^−1^ and Cu at 100 or 500 mg·kg^−1^. These concentrations were set to cover the low-to-high pollution range of actual agricultural soils, and the Cu concentrations were lower than the maximum lethal concentration for *Chlorella* (2000 mg·kg^−1^) and the maximum allowable copper content standard for agricultural soils in China [15,16].

### 2.2. Microcosm Design and Incubation

**Figure 1:**
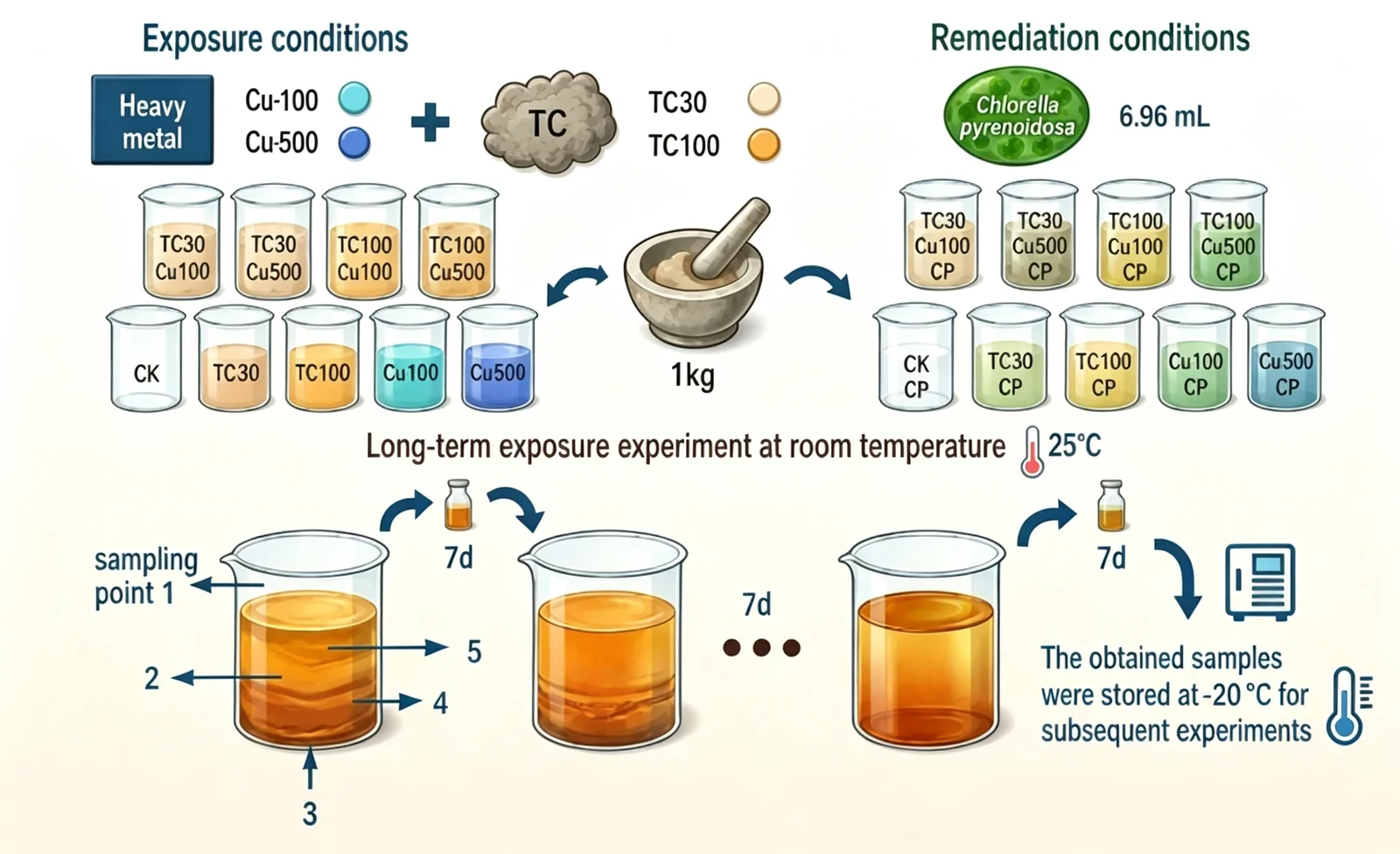
Schematic representation of the 49-d soil microcosm experiment. CP denotes supplementation with *Chlorella pyrenoidosa*.

An experiment was conducted to investigate the soil remediation effect of *Chlorella* under combined pollutant stress as summarized in Figure 1. A total of 18 experimental groups were established in culture boxes, and each box was filled with 1 kg of soil. All experimental groups were performed in triplicate. The C. pyrenoidosa biomass was uniformly mixed into the soil at a rate of 6.96 mL·kg^−1^ dry soil (equivalent to ~6.96 g dry weight·kg^−1^ soil). This rate was selected based on preliminary dose–response experiments testing 0, 3.48, 6.96, and 13.92 mL·kg^−1^ soil, in which 6.96 mL·kg^−1^ achieved the optimal balance of pollutant removal and economic cost while avoiding excessive algal accumulation or secondary contamination [17]. The groups including: CK (a control group, original soil with no pollutants), TC30 (supplemented with 30 mg of tetracycline only), TC100 (supplemented with 100 mg of tetracycline only), Cu100 (supplemented with 392.68 mg of CuSO_4_·5H_2_O only), Cu500 (supplemented with 1963.41 mg of CuSO_4_·5H_2_O only), TC30Cu100, TC30Cu500, TC100Cu100, TC100Cu500, and *Chlorella*-amended groups, CKCP (original soil with 6.96 mL of *Chlorella* suspension), TC30CP, TC100CP, Cu100CP, Cu500CP, TC30Cu100CP, TC30Cu500CP, TC100Cu100CP, and TC100Cu500CP (corresponding pollutant groups with 6.96 mL of *Chlorella* suspension). The single and combined treatment groups summarized in Figure 1 are detailed in Table 1.

All experiments were conducted simultaneously at room temperature (approximately 25 ± 2 °C). Deionized water was added every 7 days to maintain the soil moisture content at around 25% (v·m^−1^), ensuring the growth and reproduction of algal cells [18]. Every 7 days, 100 g of soil samples were collected from different positions of each treatment group and stored frozen at −20 °C.

### 2.3. Soil Physicochemical Properties and Enzyme Activities

#### 2.3.1. Physicochemical Properties

**Soil pH.** The pH was measured potentiometrically according to HJ 962-2018 [19]. Air-dried soil was mixed with distilled water at a 1:2.5 soil-to-water ratio (w·v^−1^), shaken for 30 min, allowed to stand for 30 min, and measured with a calibrated pH meter.

**Electrical conductivity.** EC was measured according to HJ 802-2016 [20]. Soil (20.00 g) was extracted with 100 mL of water at 20 ± 1 °C by shaking for 30 min. After standing for 30 min, the suspension was filtered or centrifuged and the conductivity of the extract was measured.

**Soil organic matter.** SOM was measured by potassium dichromate (K_2_Cr_2_O_7_) oxidation and ferrous sulfate titration following GB 9834-88 [21]. Soil (4.00 g) was mixed with 5 mL 0.8 mol·L^−1^ potassium dichromate and 5 mL concentrated H_2_SO_4_ and heated at 185–190 °C for 5 min. After cooling and dilution to 60–70 mL, o-phenanthroline indicator was added and the remaining dichromate was titrated with standard ammonium ferrous sulfate. A reagent blank was analysed in parallel.

(1) SOM = [C × (V_0_ − V) × 3.0 × 10^−3^ × 1.33/m] × 1000 × 1.724

where SOM is soil organic matter (mg·kg^−1^), C is the concentration of ammonium ferrous sulfate (mol·L^−1^), V_0_ and V are titrant volumes for the blank and sample (mL), respectively, and m is dry soil mass (g) [22].

#### 2.3.2. Enzyme Activities

**Dehydrogenase.** Activity was determined using triphenyltetrazolium chloride (TTC) reduction [23]. Soil (5 g) was mixed with 5 mL 0.4% TTC, incubated in the dark at 37 °C for 24 h, extracted with acetone, and centrifuged at 4000 r·min^−1^ for 5 min. Absorbance was measured at 485 nm and activity was calculated from a TTC-derived standard curve.

**Catalase.** Soil (1.0 g, <2 mm) was mixed with 10 mL phosphate buffer and equilibrated at 25 °C for 10 min. H_2_O_2_ (5 mL, 30%) was added, and the mixture was reacted in the dark for 5 min. The reaction was stopped with H_2_SO_4_, centrifuged at 4000 r·min^−1^ for 10 min, and filtered through a 0.45-µm membrane. Absorbance was measured at 240 nm.

**Nitrate reductase.** Activity was measured using the phenol disulfonic acid colorimetric procedure [24]. Frozen soil (1 g) was incubated anaerobically with CaCO_3_, KNO_3_, and glucose at 30 °C for 24 h. After extraction, filtration, and evaporation, the residue was developed with phenol disulfonic acid and quantified against a nitrate-N standard curve. Exact reagent concentrations and the final measurement wavelength should be reported for reproducibility [24].

### 2.4. TC Dissipation and Extractable Cu

#### 2.4.1. Tetracycline Extraction and HPLC Analysis

A sieved soil sample (1.0–2.0 g, weighed to the nearest 0.1 g) was transferred into a 50 mL centrifuge tube and extracted with 20 mL of Na_2_EDTA–McIlvaine–methanol solution by shaking for 20 min, followed by centrifugation at 3500 r·min^−1^ for 10 min. This extraction process was repeated twice, and the resulting supernatants were combined and diluted to a final volume of 50 mL. For solid-phase extraction (SPE) purification, a 5 mL aliquot of the supernatant was loaded onto a pre-conditioned HLB cartridge at a flow rate of 1.0 mL·min^−1^, rinsed with 5 mL of a methanol–water solution (5:95, *v*/*v*), and subsequently dried under vacuum. The target analytes were eluted with 5 mL of an oxalic acid–methanol solution, and the eluate was concentrated to less than 0.5 mL under a gentle stream of nitrogen at 40 °C. The residue was then reconstituted in 2 mL of the oxalic acid–methanol solution and passed through a 0.22 μm membrane filter for high-performance liquid chromatography (HPLC) analysis. Chromatographic separation was achieved on an Agilent Zorbax Eclipse Plus C18 column (250 mm × 4.6 mm, 4.6 μm; Agilent Technologies, Wilmington, DE, USA) maintained at 30 °C, with an injection volume of 20 μL and a detection wavelength of 355 nm [25].

#### 2.4.2. Extractable Copper Extraction and ICP-MS Analysis

Available copper in soil was extracted using the universally accepted DTPA (diethylenetriaminepentaacetic acid) method and quantified via inductively coupled plasma mass spectrometry (ICP-MS). Briefly, a 10.0 g aliquot of sieved, air-dried soil was weighed into a 50 mL polypropylene centrifuge tube, followed by the addition of 20 mL of a DTPA extraction solution containing 0.005 M DTPA, 0.01 M CaCl_2_, and 0.1 M triethanolamine (TEA), adjusted to a precise pH of 7.30 ± 0.05. The soil-solution mixture was shaken continuously on a reciprocal shaker at 180 rpm for 2 h at a constant temperature of 25 °C. Subsequently, the suspension was centrifuged at 4000 rpm for 15 min and filtered through a 0.45 μm cellulose acetate membrane filter. The obtained filtrate was acidified with 2% (*v*/*v*) ultra-pure nitric acid HNO_3_ prior to instrumental analysis. The concentration of available Cu was determined using an ICP-MS instrument (Agilent 7800; Agilent Technologies, Wilmington, DE, USA), operating with an RF power of 1550 W, a nebulizer gas flow rate of 1.05 L·min^−1^, and using Rhodium (Rh) as an internal standard to compensate for matrix effects [26,27].

### 2.5. Metagenomic Sequencing and Annotation

Metagenomic sequencing was conducted on the Illumina NovaSeq 6000 platform (Illumina, San Diego, CA, USA) in this study. Raw data were quality-controlled using Trimmomatic (v0.39) software to remove adapters and low-quality reads (with N content > 10% or bases with Q < 10 accounting for >50%), yielding high-quality clean data. Sequence assembly was performed using MEGAHIT (v1.2.9) software based on the De Bruijn graph algorithm, and contigs longer than 500 bp were selected for subsequent analyses. Open Reading Frames (ORFs) were predicted via Prodigal (v2.6.3) software and translated into amino acid sequences. Redundancy reduction was carried out using CD-HIT (v4.8.1) software (identity = 95%, coverage = 90%) to construct a non-redundant gene set, and the TPM (Transcripts Per Million) abundance of genes was calculated through bowtie2 (v2.4.5) alignment. For species annotation, genes were aligned against the NR database using DIAMOND (v2.0.15) software, and taxonomic information was determined by combining it with the LCA (Lowest Common Ancestor) algorithm. Community structure analysis included abundance statistics from the phylum to species levels, enabling a comprehensive characterization of the composition and differential features of the microbial community [28].

### 2.6. Statistical Analysis

Figures were prepared in Origin 2023. Heat maps were produced in HemI v1.0, and redundancy analysis (RDA) was conducted in Canoco v5.0. LEfSe was used for exploratory differential-taxon analysis. Spearman correlations with |r| > 0.5 and *p* < 0.05 were used to construct co-occurrence networks. For treatment-response variables measured through time, an appropriate final analysis should include TC, Cu, algal amendment, time, and their interactions, with microcosm identity treated as a random effect. Multiple-testing correction should be applied to pairwise comparisons and network edges. Means, standard deviations, exact biological replicate numbers, test statistics, degrees of freedom, and adjusted *p* values should be reported.

In this study, Origin 2023 was used to plot the curves of basic soil physicochemical properties, bar charts of enzyme contents, and mitigation efficiency graphs; under the action of *Chlorella*, circularity analysis for visualizing microbial community structure was performed using an online tool. The linear discriminant analysis (LDA) effect size (LEfSe) method was employed to analyze changes in the microbial community induced by TC/Cu addition [29]. HemI v. 1.0 and Canoco v. 5.0 were used for heatmap generation and redundancy analysis (RDA), respectively. Network analysis based on Spearman’s rank correlation was conducted to explore the relationships between potential driving factors (including microbial community structure with relative abundance at the genus level showing a correlation coefficient > 0.5 and *p* < 0.05) and antibiotic resistance genes, as well as environmental factors. All experiments in this study were performed in triplicate. Analysis of variance (ANOVA) was conducted using SPSS 21.0 software [30].

## 3. Results and Discussion

### 3.1. TC Dissipation and Decrease in Extractable Cu

**Figure 2:**
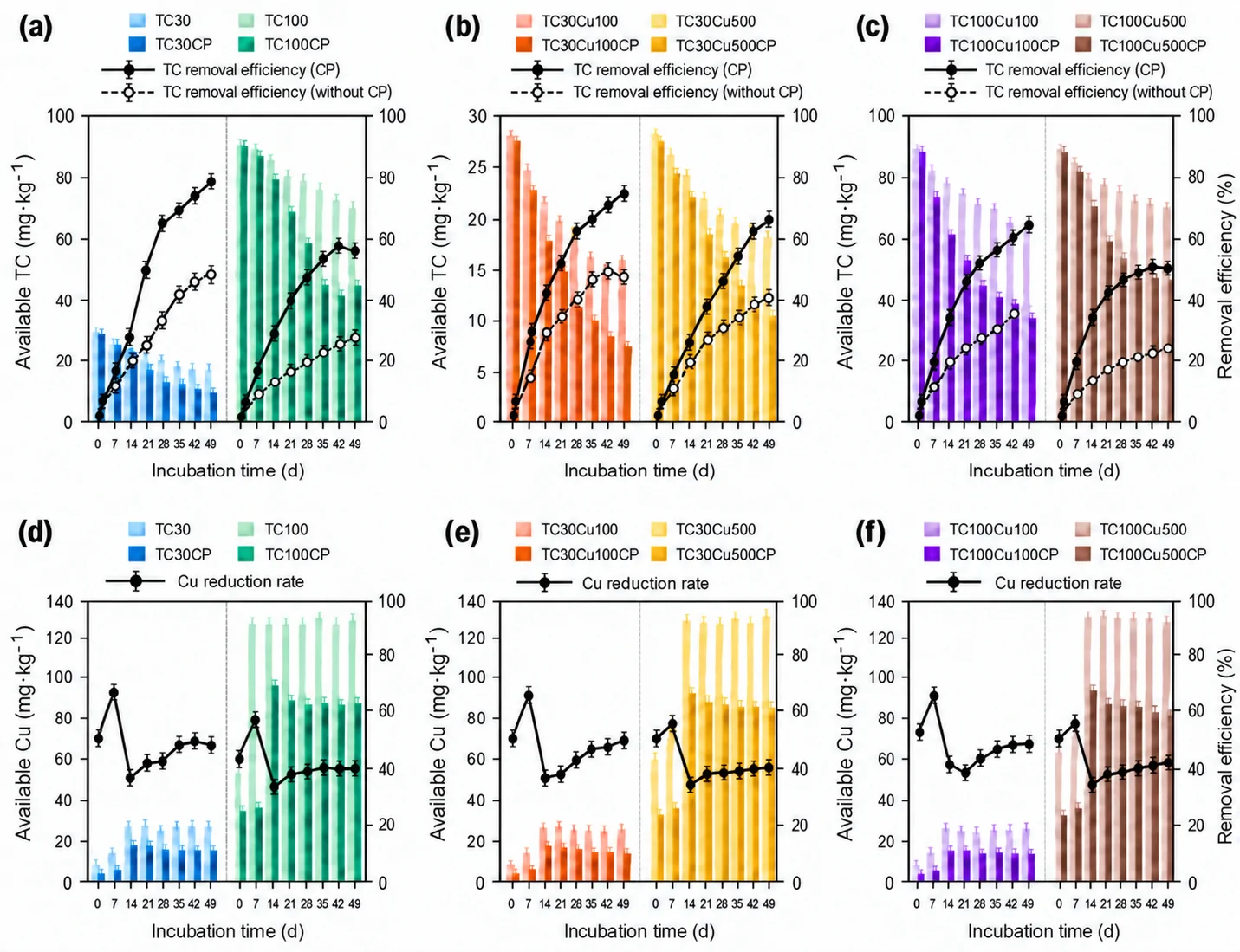
Changes in parent tetracycline (TC) and extractable copper (Cu) during the 49-d incubation: (**a**–**c**) residual TC concentration and apparent removal; (**d**–**f**) extractable Cu concentration and percentage decrease. CP denotes *Chlorella pyrenoidosa* supplementation.

The results showed a significantly positive effect of *C. pyrenoidosa* (CP) amendment on TC dissipation, with residual TC concentrations markedly lower in CP-amended treatments than in unamended controls (Figure 2a–c).

In the absence of *Chlorella pyrenoidosa* (CP), TC dissipation ranged from 22.7% to 43.5%, whereas CP amendment significantly increased the dissipation efficiency to 49.9–73.5% (*p* < 0.05). TC dissipation in the unamended soils was likely governed by multiple processes, including degradation by indigenous microorganisms capable of utilizing TC as a carbon or nitrogen source or transforming it through cometabolism [31], abiotic photolysis [32], and, in the co-contaminated soils, Cu–TC complexation and the associated changes in TC extractability and redistribution [15]. In soils contaminated with TC alone, the enhanced TC dissipation following CP amendment may be attributed to the combined effects of TC sorption onto algal cells and the indirect stimulation of the degradative activity of indigenous microorganisms [33,34]. Notably, the CP-amended TC–Cu co-contaminated treatments exhibited significantly greater TC dissipation than the corresponding single-TC treatments; for example, the dissipation efficiency reached 73.5% in TC30Cu100CP compared with 69.7% in TC30CP (*p* < 0.05). This response is consistent with the findings of Li et al. [35], who reported that low concentrations of heavy metals may stimulate microbial stress responses and the expression of catabolic enzymes, thereby promoting antibiotic transformation in soil. By analogy with the microelectrochemical enhancement of TC degradation in soil reported for microplastics [36], Cu may have modified electron-transfer or redox conditions at soil mineral–organic interfaces, thereby facilitating TC transformation and stimulating the activity of TC-degrading microorganisms.

The temporal changes in bioavailable Cu concentrations and the corresponding reduction efficiencies are summarized in Figure 2d–f. CP amendment reduced bioavailable Cu by 35.6% in Cu500CP to 50.6% in TC100Cu100CP, with values significantly higher than those observed in the corresponding unamended treatments (*p* < 0.05). Consistent with the pattern of TC dissipation, the reduction in bioavailable Cu was greater in the co-contaminated treatments than in the single-Cu treatments, as demonstrated by the values of 48.2% in TC30Cu100CP versus 44.5% in Cu100CP and 40.0% in TC100Cu500CP versus 35.6% in Cu500CP. This enhancement may be associated with TC–Cu complexation, which could alter Cu speciation and its affinity for algal biomass, microorganisms, and soil mineral surfaces, thereby facilitating Cu immobilization in CP-amended soils [34]. Across the contaminated treatments, bioavailable Cu concentrations initially increased and subsequently reached a plateau, stabilizing at 25.2 mg·kg^−1^ in Cu100 and 132.1 mg·kg^−1^ in Cu500; this temporal pattern was likely associated with the redistribution of Cu among soil fractions and changes in its chemical speciation [37]. The CP-mediated reduction in bioavailable Cu was characterized by a rapid initial phase followed by gradual stabilization. The reduction efficiency was significantly higher under the lower Cu loading in Cu100CP than under the higher Cu loading, possibly because excessive Cu imposed physiological stress on *C. pyrenoidosa*, thereby suppressing its metabolic activity and Cu-binding capacity. The enhanced reduction in bioavailable Cu observed in the co-contaminated treatments was likely related to the synergistic interactions between TC and Cu. Specifically, TC–Cu complexation may have modified Cu lability and favored its retention by algal biomass and associated microbial or mineral surfaces. In addition, TC-induced changes in soil physicochemical conditions and microbial activity may have created a microenvironment favorable for CP-associated Cu immobilization.

The observed enhancement of TC dissipation and Cu immobilization by CP supplementation can be mechanistically linked to several intrinsic biological properties of *C. pyrenoidosa*. Its cell surface, rich in functional groups, facilitates adsorption of both TC and Cu^2+^ [33]; its intracellular enzymatic systems enable metabolic transformation of tetracycline [12]; and its secretion of extracellular polymeric substances (EPS) promotes metal chelation and may stimulate algal–microbial interactions that enhance co-metabolic degradation by indigenous bacteria [12,33]. Collectively, these properties—surface adsorption, intracellular biotransformation, and EPS-mediated interspecies interactions—underpin the dual remediation capacity of CP in TC–Cu co-contaminated soils.

### 3.2. Soil Physicochemical Properties

**Figure 3:**
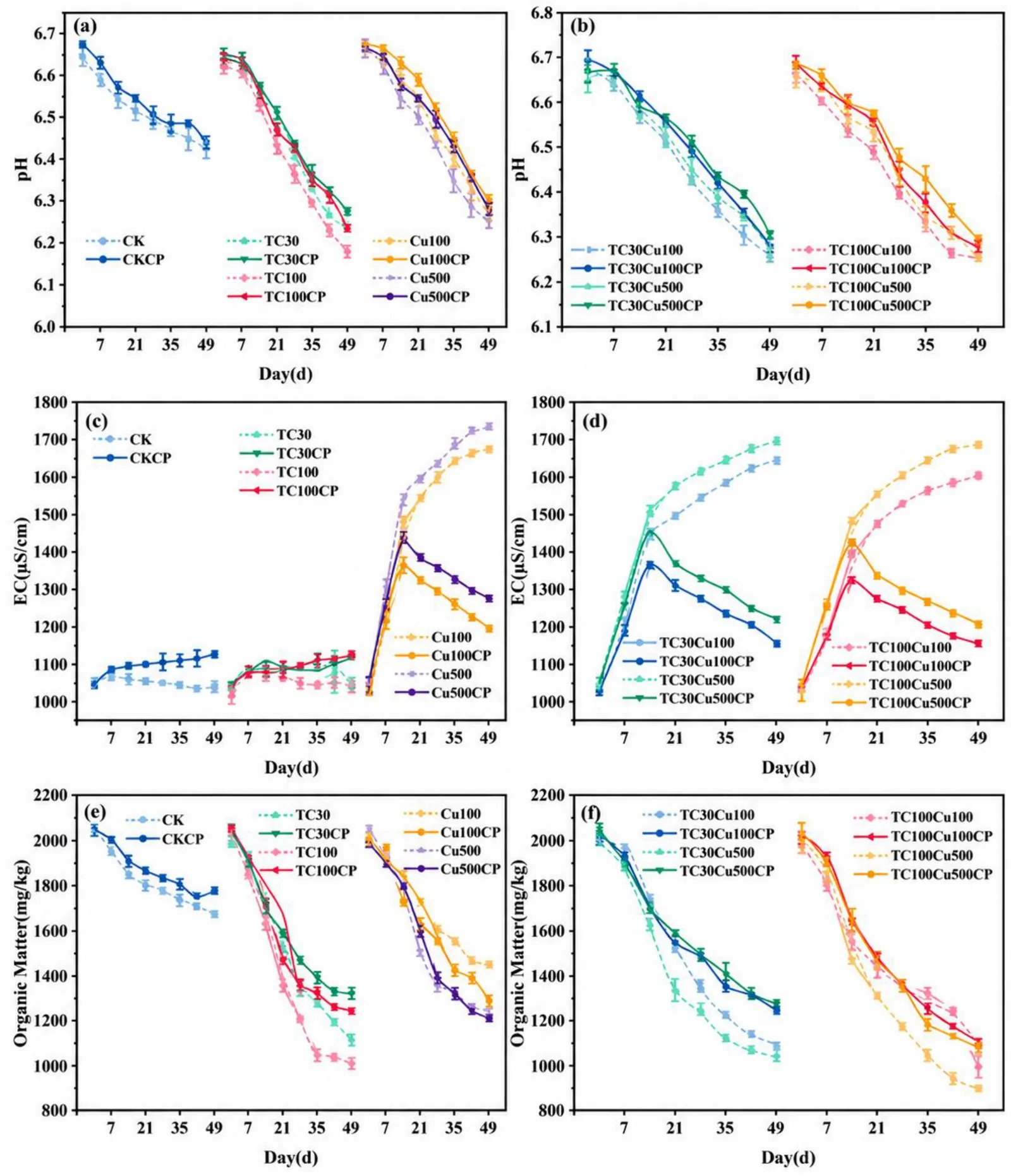
Temporal changes in (**a**,**b**) soil pH, (**c**,**d**) electrical conductivity, and (**e**,**f**) soil organic matter in unamended and *Chlorella pyrenoidosa*-amended microcosms.

Temporal changes in soil pH in the contaminated and CP-amended treatments are shown in Figure 3a and Figure 3b, respectively. Among the single-contaminant treatments, the measured pH values decreased by 6.25%, 6.27%, 7.13%, and 7.65% in TC30, TC100, Cu100, and Cu500, respectively. In the TC–Cu co-contaminated treatments, the corresponding decreases ranged from 6.82% to 7.05%, intermediate between those observed in the single-contaminant treatments and therefore providing no evidence of a synergistic acidification effect. This response may be partly associated with TC–Cu complexation, which can decrease the activity of free Cu^2+^ and consequently attenuate its influence on soil pH. Relative to the corresponding unamended treatments, CP amendment resulted in modest increases in soil pH: pH increased by 3.2% and 3.3% in TC30CP and TC100CP, respectively, and by 2.4% and 3.3% in Cu100CP and Cu500CP, respectively. The greatest increase, 5.8%, was observed in the high-concentration co-contaminated treatment TC100Cu500CP. Previous studies have suggested that extracellular polymeric substances (EPS) released by *Chlorella* can limit net H^+^ release [38]. Therefore, EPS production and other CP-associated biological processes may have buffered the acidification induced by TC and Cu^2+^, thereby creating soil conditions more favorable for contaminant attenuation.

Temporal changes in soil EC in the contaminated and CP-amended treatments are presented in Figure 3c and Figure 3d, respectively. No significant changes in EC were detected in TC30 or TC100 during the incubation period (*p* > 0.05). By contrast, EC increased to 1675 μS·cm^−1^ in Cu100 and 1740 μS·cm^−1^ in Cu500. The co-contaminated treatments exhibited trends similar to those of the corresponding single-Cu treatments. The final EC values of TC30Cu100 and TC100Cu100 both reached approximately 1620 μS·cm^−1^, representing a 55.8% increase relative to CK but a 3.3% decrease relative to Cu100. Similarly, EC reached approximately 1700 μS·cm^−1^ in TC30Cu500 and TC100Cu500, corresponding to an increase of approximately 63.5% relative to CK and a decrease of 1.3% relative to Cu500. Compared with the corresponding unamended treatments, CP amendment decreased EC by 28.1% in Cu100CP, 27.0% in Cu500CP, and 27.4–29.7% in the co-contaminated treatments, with the greatest reduction observed in TC100Cu100CP. In the Cu-containing treatments, the initial increase in EC coincided with an increase in bioavailable Cu, whereas the subsequent decline broadly paralleled the CP-associated reduction in bioavailable Cu. However, because EC reflects the total concentration and mobility of soluble ions rather than Cu alone, this parallel temporal pattern should be interpreted as an association rather than evidence of a direct causal relationship. Overall, Cu contamination significantly increased soil EC, whereas EC in the single-TC treatments remained comparable to that in CK. CP amendment significantly reduced soil EC, consistent with the findings of Hee et al. [39].

Temporal changes in SOM in the contaminated and CP-amended treatments are illustrated in Figure 3e and Figure 3f, respectively. In the single-TC treatments, SOM decreased by 41.3–50.2%, whereas the corresponding decreases were limited to 14.9–15.2% following CP amendment. In the single-Cu treatments, the decrease in SOM after CP amendment became smaller as the Cu concentration increased, amounting to 11.2% in Cu100CP and 2.8% in Cu500CP. This concentration-dependent pattern may reflect the combined effects of algal-derived organic inputs and the suppression of microbial SOM mineralization under elevated Cu stress. Previous studies have indicated that *Chlorella* amendment can improve the properties of Cu-contaminated soils and contribute to the preservation or accumulation of SOM through algal biomass and extracellular organic substances [40]. Following CP amendment, SOM in the TC–Cu co-contaminated treatments increased significantly by 112–232 mg·kg^−1^. Rather than directly demonstrating TC–Cu complexation, this increase may reflect the input of algal-derived organic matter and the interactions of algal cells or EPS with TC and Cu, which can alter their speciation, retention, and biological effects in soil [34]. Leonard et al. demonstrated that CP application significantly increased soil microbial abundance [31]. In addition, CP may stimulate microbial growth and proliferation [41], thereby increasing the contribution of microbial biomass and residues to the SOM pool. Collectively, the accumulation of algal biomass, EPS, and microbial residues may have promoted SOM retention and created conditions favorable for contaminant attenuation.

### 3.3. Soil Enzyme Activities

#### 3.3.1. Dehydrogenase

**Figure 4:**
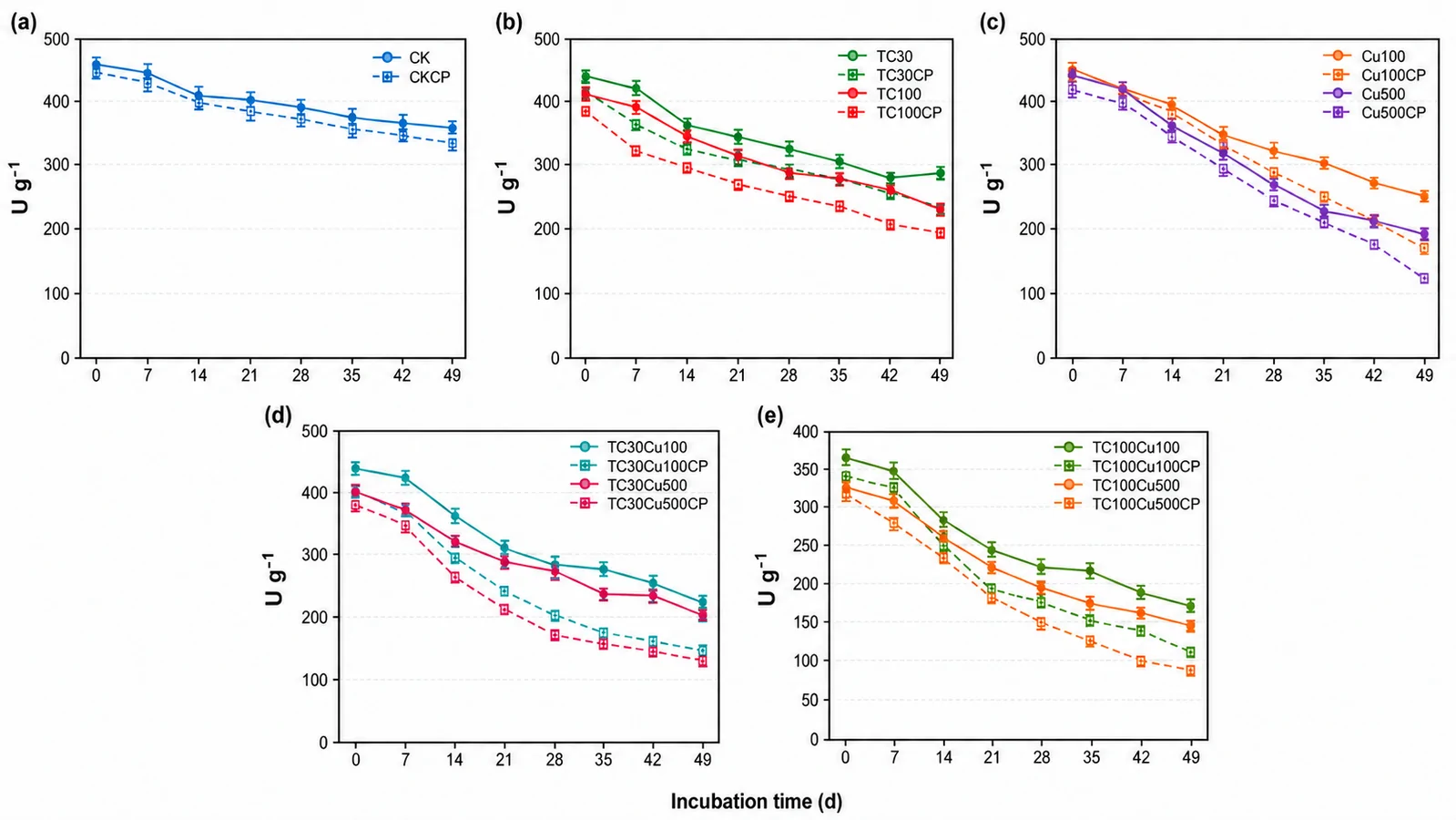
Soil dehydrogenase activity during the 49-d incubation: (**a**) control group; (**b**) single tetracycline treatments; (**c**) single copper treatments; (**d**) combined TC–Cu treatments at low TC level; (**e**) combined TC–Cu treatments at high TC level. CP denotes *Chlorella pyrenoidosa* supplementation.

The effects of TC and Cu contamination and CP supplementation on soil dehydrogenase activity are shown in Figure 4. Compared with CK, soil dehydrogenase activity decreased by approximately 28.4% in TC30 and 42.8% in TC100 at the end of the incubation period, indicating that TC markedly inhibited soil microbial oxidative metabolism. Because soil dehydrogenases are predominantly intracellular enzymes associated with viable microbial cells, the observed inhibition was more likely caused by TC-induced suppression of microbial growth, respiration, and intracellular electron-transfer processes than by direct competition between TC and enzyme substrates for catalytic sites. In the Cu-treated soils, dehydrogenase activity decreased by 51.2% and 64.9% in Cu100 and Cu500, respectively, demonstrating a concentration-dependent inhibitory effect of Cu. Dominika et al. suggested that Cu^2+^ can interact with essential enzyme functional groups, disrupt cellular integrity, and impair microbial metabolism, collectively resulting in a pronounced reduction in soil dehydrogenase activity [42]. Co-exposure to TC and Cu caused greater inhibition than either contaminant alone, with the largest decrease of 70.7% observed in TC100Cu500. This result indicates intensified biological stress under co-contamination. Following CP amendment, soil dehydrogenase activity generally increased across the treatments. Dehydrogenase activity in CKCP was 5.9% higher than that in CK, suggesting that CP amendment itself modestly stimulated soil microbial oxidative metabolism. Compared with the corresponding unamended treatments, dehydrogenase activity increased by 22.8% in TC30CP and by 15.5% in TC100CP. The greater recovery observed under the lower TC loading may reflect the relatively weaker stress imposed on soil microorganisms by low TC concentrations [43], allowing CP-associated processes to more effectively promote microbial functional recovery. These processes may include the addition of algal-derived organic substrates, modification of soil physicochemical conditions, and attenuation of the bioavailable contaminant fraction. Nevertheless, dehydrogenase activity alone cannot distinguish direct stimulation by CP from indirect effects mediated through changes in microbial biomass, soil pH, SOM, or contaminant bioavailability.

#### 3.3.2. Catalase

**Figure 5:**
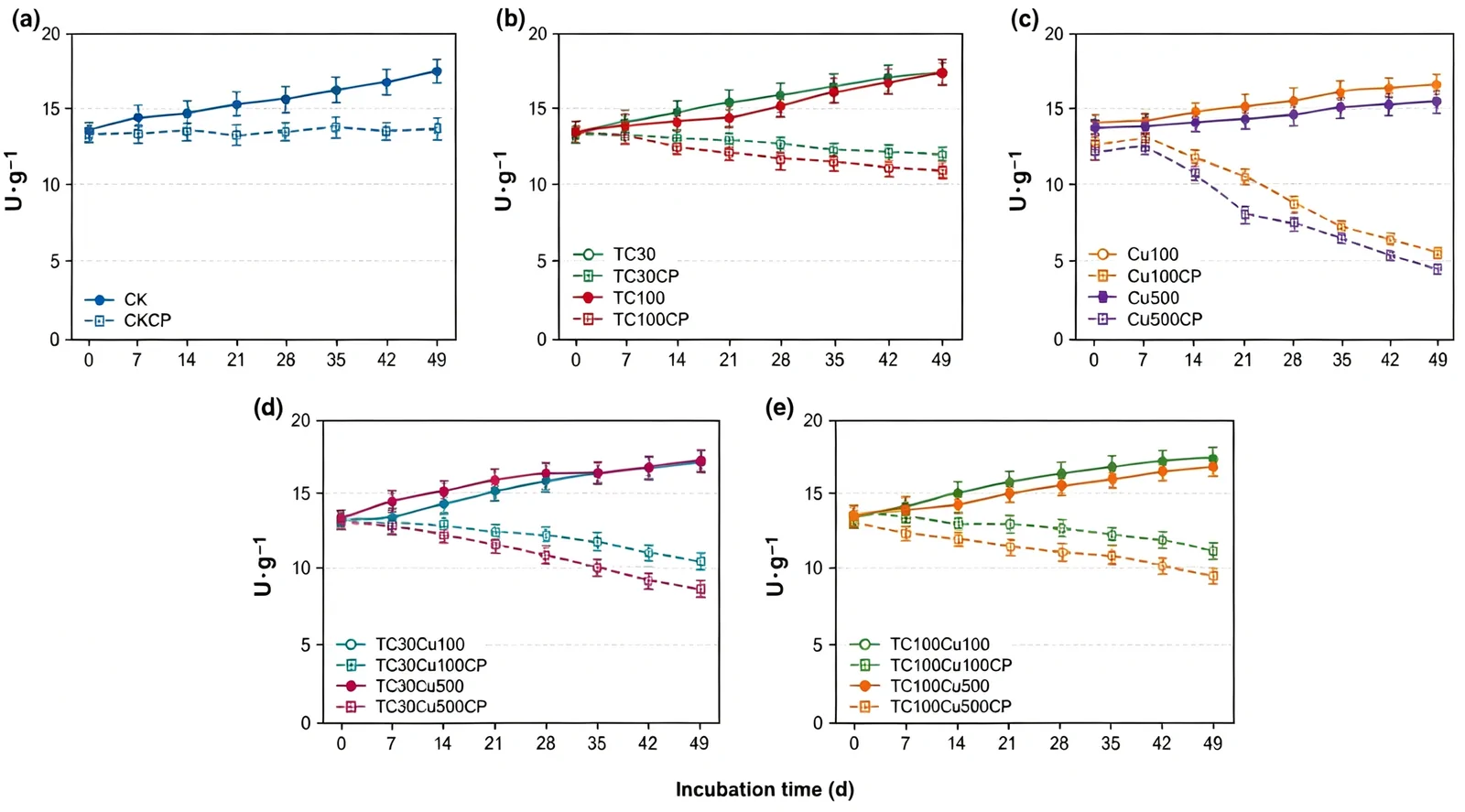
Soil catalase activity during the 49-d incubation: (**a**) control group; (**b**) single tetracycline treatments; (**c**) single copper treatments; (**d**) combined TC–Cu treatments at low TC level; (**e**) combined TC–Cu treatments at high TC level. CP denotes *Chlorella pyrenoidosa* supplementation.

Temporal changes in soil catalase (S-CAT) activity across the different treatments are presented in Figure 5. S-CAT activity in CK remained relatively stable throughout the incubation period, with a slight increase of approximately 2.9%, indicating that no appreciable loss of enzyme activity occurred in the absence of contaminant stress. The magnitude of inhibition differed markedly among contaminant types. In the single-TC treatments, S-CAT activity decreased by approximately 8.4% in TC30 and 17.3% in TC100, indicating a concentration-dependent but relatively moderate inhibitory effect of TC. By contrast, Cu caused substantially greater inhibition, with S-CAT activity decreasing by 56.1% in Cu100 and 61.5% in Cu500. In the TC–Cu co-contaminated treatments, the decreases ranged from 18.0% to 34.3%, intermediate between those observed under single-TC and single-Cu contamination. Overall, Cu exerted a stronger inhibitory effect on S-CAT activity than TC, and the magnitude of inhibition increased with Cu loading [44,45]. Because catalase is involved in the decomposition of H_2_O_2_, the pronounced decline under Cu stress may reflect both direct enzyme inhibition and disruption of microbial oxidative-stress defenses. The weaker inhibition observed in the co-contaminated treatments than in the corresponding single-Cu treatments may be partly associated with TC–Cu complexation, which can alter Cu speciation and reduce the activity of free Cu^2+^. Following *Chlorella pyrenoidosa* (CP) amendment, S-CAT activity partially recovered in all contaminated treatments. Relative to the corresponding unamended treatments, S-CAT activity increased by approximately 28% in the single-TC treatments and by 11.8–17.3% in the single-Cu treatments. A greater recovery of 23.1–31.5% was observed in the co-contaminated treatments, with TC30Cu100CP exhibiting the largest increase of 31.5%. These results indicate that CP amendment alleviated contaminant-induced inhibition of S-CAT activity, although the smaller recovery under single-Cu contamination suggests that Cu imposed more persistent biological stress than TC. The recovery of S-CAT activity may have resulted from a combination of reduced contaminant bioavailability, improved soil physicochemical conditions, and enhanced microbial activity rather than from direct stimulation of catalase by CP alone. This CP-associated restoration of soil catalase activity is consistent with the findings of Li et al. [43].

#### 3.3.3. Nitrate Reductase

**Figure 6:**
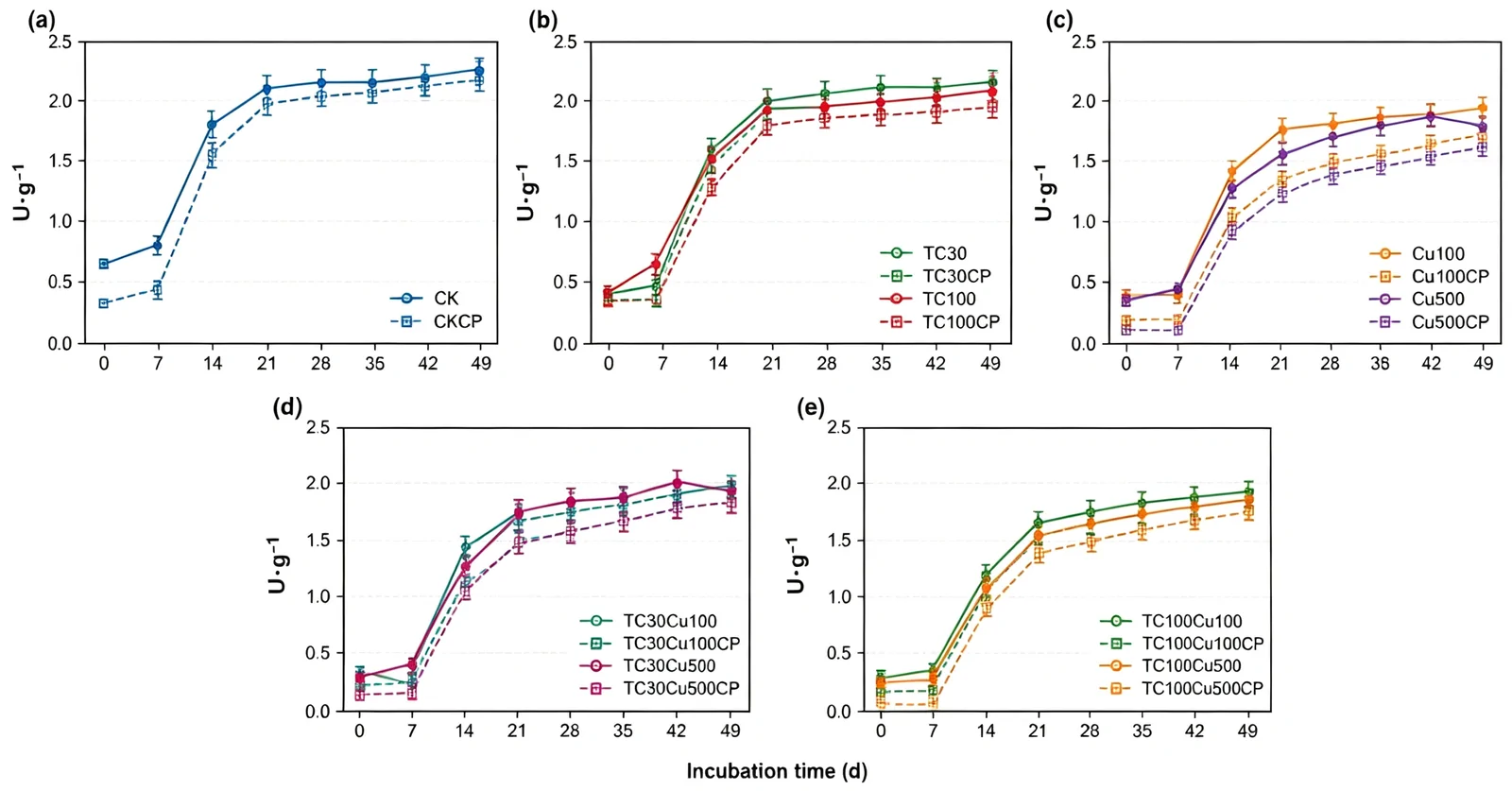
Soil nitrate reductase activity during the 49-d incubation: (**a**) control group; (**b**) single tetracycline treatments; (**c**) single copper treatments; (**d**) combined TC–Cu treatments at low TC level; (**e**) combined TC–Cu treatments at high TC level. CP denotes *Chlorella pyrenoidosa* supplementation.

Temporal changes in soil nitrate reductase (NR) activity across the different treatments are shown in Figure 6. In CK, NR activity increased from 0.353 to 2.132 over the incubation period, corresponding to an increase of approximately 504.0% and indicating a marked enhancement of microbial nitrate-reduction potential in the uncontaminated soil. By contrast, NR activity in all contaminated treatments remained significantly lower than that in CK. Among the contaminants, Cu exerted a stronger inhibitory effect on NR activity than TC. This difference may reflect Cu-induced oxidative stress, damage to microbial cell membranes, and interference with enzyme functional groups and cofactor-dependent electron-transfer processes, whereas TC primarily suppresses microbial growth and protein synthesis and thereby indirectly restricts nitrate-reducing metabolism. Consistent with the partial recovery observed for S-CAT activity, *CP* amendment slightly increased NR activity in all contaminated treatments. Relative to the corresponding unamended treatments, NR activity increased by 2.4–10.4%, with Cu100CP exhibiting the greatest recovery. Algal-derived organic compounds and other metabolites may modify carbon and nitrogen availability, soil pH, and interactions among nitrogen-cycling microorganisms, thereby indirectly promoting microbial nitrate-reduction processes [46]. In addition, the sorption and immobilization of Cu by CP cells and extracellular polymeric substances may decrease the activity of free Cu^2+^ and alleviate its toxicity toward nitrate-reducing microbial communities. Collectively, these processes may explain the partial recovery of NR activity following CP amendment.

### 3.4. Tetracycline Resistance Genes and Microbial Community Composition

#### 3.4.1. Tetracycline Resistance Genes

**Figure 7:**
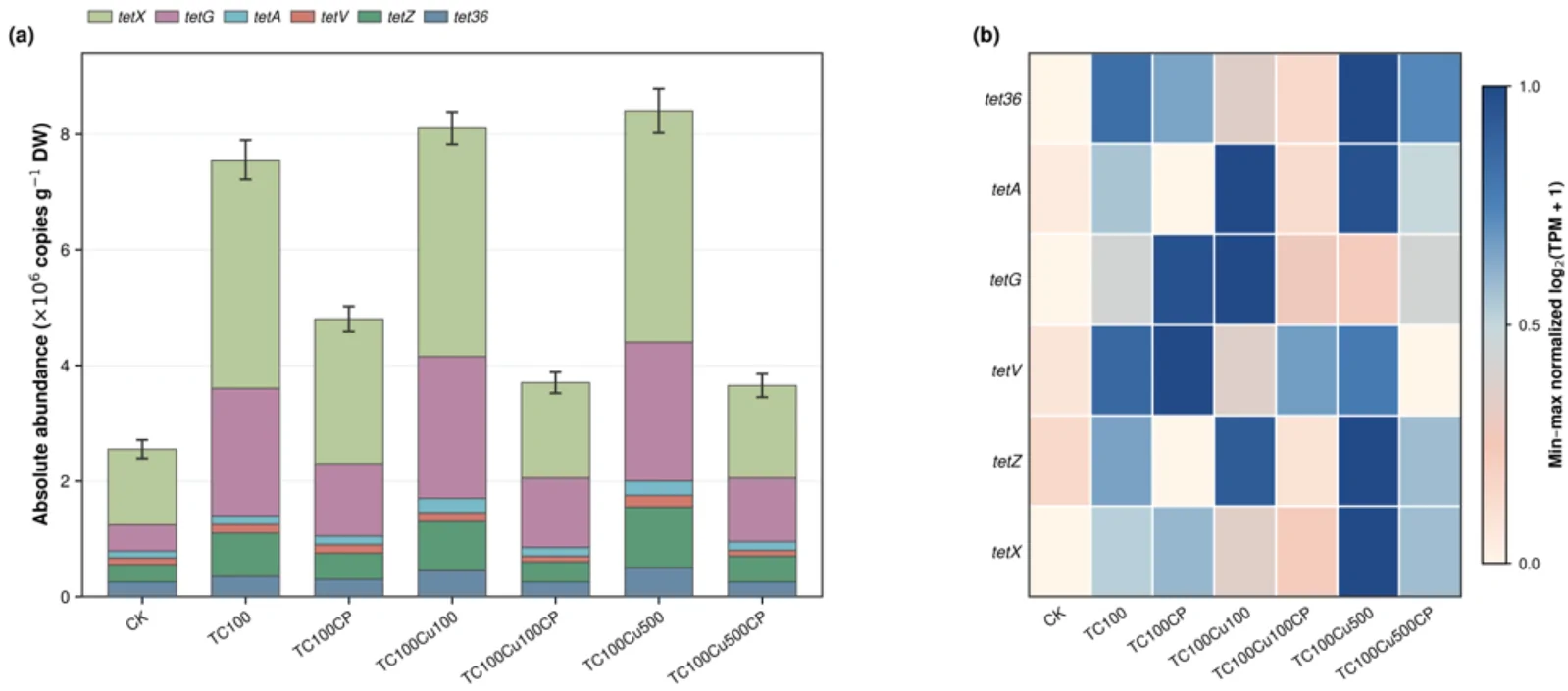
Reported tetracycline resistance gene profiles in selected treatments: (**a**) gene-level abundance and (**b**) normalized heat map.

The absolute abundances and composition of the six detected TRGs are presented in Figure 7a, including *tet36*, *tetV*, *tetZ*, *tetG*, *tetA*, and *tetX*. Total TRG abundance ranged from approximately 2.5 × 10^8^ copies·g^−1^ DW in CK to 8.4 × 10^8^ copies·g^−1^ DW in TC100Cu500. Compared with CK, TC100 markedly increased total TRG abundance to approximately 7.5 × 10^8^ copies·g^−1^ DW, indicating strong selection for tetracycline resistance following TC exposure. This observation is consistent with Cheng et al. [47], who reported that environmental TC exposure can select for and enrich TRGs. Cu co-contamination further increased total TRG abundance to approximately 8.1 × 10^8^ copies·g^−1^ DW in TC100Cu100 and 8.4 × 10^8^ copies·g^−1^ DW in TC100Cu500. The progressive increase with Cu loading suggests that Cu intensified the selective pressure imposed by TC. Notably, the increase in TRG abundance occurred despite the enhanced TC dissipation previously observed under low-Cu conditions, indicating that contaminant dissipation did not necessarily result in a corresponding reduction in resistance genes. Following *Chlorella pyrenoidosa* (CP) amendment, total TRG abundance decreased to approximately 4.8 × 10^8^ copies·g^−1^ DW in TC100CP, 3.7 × 10^8^ copies·g^−1^ DW in TC100Cu100CP, and 3.6 × 10^8^ copies·g^−1^ DW in TC100Cu500CP. These values correspond to approximate reductions of 36%, 54%, and 57%, respectively, relative to the corresponding unamended treatments. The greatest absolute decrease, approximately 4.8 × 10^8^ copies·g^−1^ DW, was observed in TC100Cu500CP. The CP-associated reduction in total TRG abundance may have resulted from decreased contaminant bioavailability, shifts in the abundance of TRG-carrying microorganisms, and changes in environmental conditions affecting horizontal gene transfer. The min–max-normalized log_2_ (TPM + 1) profiles of the six TRGs across the treatments are shown in Figure 7b. According to the absolute-abundance results in Figure 7a, *tetX* was the predominant detected TRG and accounted for the largest proportion of total TRG abundance across the treatments, with an estimated abundance of approximately 1.2–4.0 × 10^8^ copies·g^−1^ DW. The predominance of tetX may be related to its distinct resistance mechanism, as it encodes a flavin-dependent monooxygenase capable of enzymatically modifying and inactivating tetracyclines [48,49]. Among the efflux-associated genes, *tetG* was the most abundant, whereas *tetA*, *tetV*, and *tetZ* occurred at comparatively lower levels. By contrast, *tet36*, which encodes a ribosomal-protection determinant, represented a relatively small proportion of total TRG abundance. The normalized profiles further indicate that CP amendment did not affect all individual TRGs uniformly. Although CP substantially decreased total absolute TRG abundance, the relative patterns of individual genes varied among the TC-only and TC–Cu treatments. These gene-specific responses may have been governed by differences in microbial hosts, resistance mechanisms, genetic mobility, and contaminant-induced co-selection.

#### 3.4.2. Microbial Community Composition

**Figure 8:**
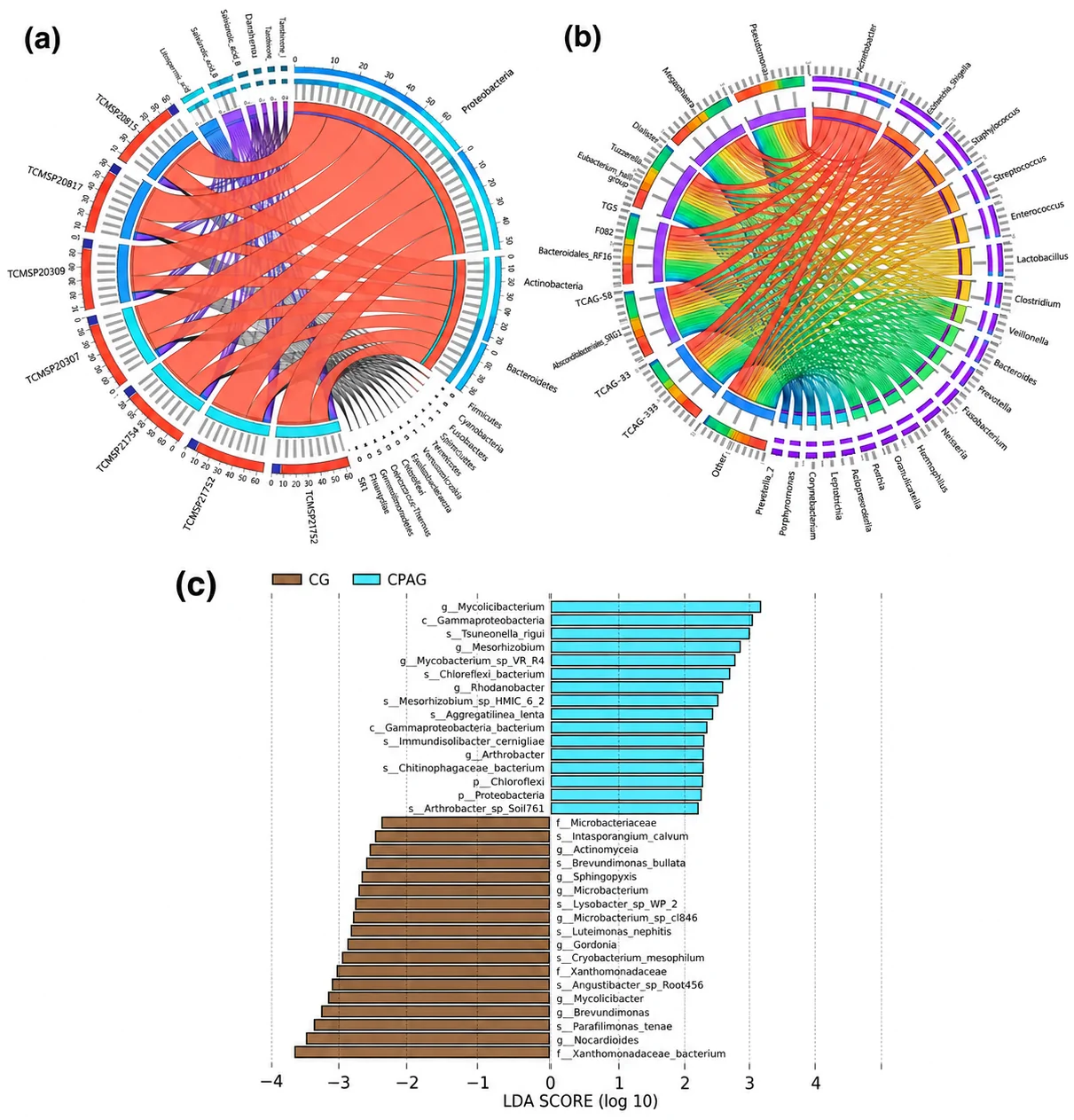
Microbial community composition and exploratory differential-taxon analysis: (**a**) phylum-level profile, (**b**) genus-level profile, and (**c**) LEfSe comparison of pooled contaminated (CG) and *Chlorella pyrenoidosa*-amended (CPAG) samples.

The distributions of the 20 most abundant bacterial phyla across the treatments are summarized in Figure 8a. Proteobacteria and Actinobacteria dominated the bacterial communities, collectively accounting for approximately 80–85% of the total relative abundance. The predominance of Proteobacteria may reflect their broad metabolic versatility, rapid response to environmental disturbance, and frequent occurrence among contaminant-tolerant and antibiotic-resistant bacterial populations [50]. Relative to CK, the relative abundance of Proteobacteria increased by 7.9–9.5% in the contaminated treatments, with the greatest increase observed in TC100Cu500. CP amendment subsequently decreased its relative abundance by 3.5–5.1% compared with the corresponding unamended treatments. Firmicutes and Bacteroidetes also increased following contaminant exposure, whereas CP amendment reduced their relative abundances by 9.4–21%. These phyla have frequently been reported as potential reservoirs of TRGs in contaminated soils [4]. Cu addition was associated with a decrease in Gemmatimonadetes relative to the corresponding single-TC treatments, suggesting that members of this phylum may have been sensitive to Cu stress. Notably, CP amendment significantly increased the relative abundances of Desulfobacterota and Chloroflexi (*p* < 0.05). In TC100Cu100CP, their abundances were 12.3% and 15.6% higher, respectively, than those in TC100Cu100. The distributions of the 20 most abundant bacterial genera are shown in Figure 8b, among which *Rhodanobacter*, *Lysobacter*, *Mycobacterium*, and *Chloroflexus* were prominent. The relative abundance of *Rhodanobacter* varied markedly among treatments and reached its maximum in TC100Cu500, where it was approximately 14.9% higher than that in CK. This pattern indicates that *Rhodanobacter* responded strongly to TC–Cu co-contamination. Certain members of *Rhodanobacter* have been associated with contaminant tolerance and antibiotic-efflux determinants [4]. LEfSe analysis with an LDA-score threshold of 2.5 identified the taxa that discriminated between the contaminated groups (CG) and CP-amended groups (CPAG) (Figure 8c). CG was characterized by the enrichment of Microbacteriaceae, Actinomycetia, *Sphingopyxis*, *Microbacterium*, *Lysobacter* sp. WF2, *Luteimonas mephitis*, *Gordonia*, Xanthomonadaceae, *Brevundimonas*, and *Nocardioides*. By contrast, CPAG was enriched in *Mycolicibacterium*, Gammaproteobacteria, *Mesorhizobium*, *Rhodanobacter*, *Arthrobacter*, Chloroflexi, and Proteobacteria, together with species-level biomarkers such as *Mycobacterium* sp. YC_RL4 and *Arthrobacter* sp. Soil763. Although *Rhodanobacter* reached its highest abundance in TC100Cu500, its identification as a CPAG-associated biomarker in the pooled LEfSe comparison indicates a treatment-dependent response rather than a simple monotonic decline following CP amendment. Therefore, *Rhodanobacter* and *Arthrobacter* should be classified as CPAG-associated biomarkers, whereas *Luteimonas mephitis* and *Lysobacter* sp. WF2 were associated with CG. The enrichment of Chloroflexi in CPAG indicates substantial restructuring of the bacterial community following CP amendment.

#### 3.4.3. Associations Among TRGs and Microbial Taxa

**Figure 9:**
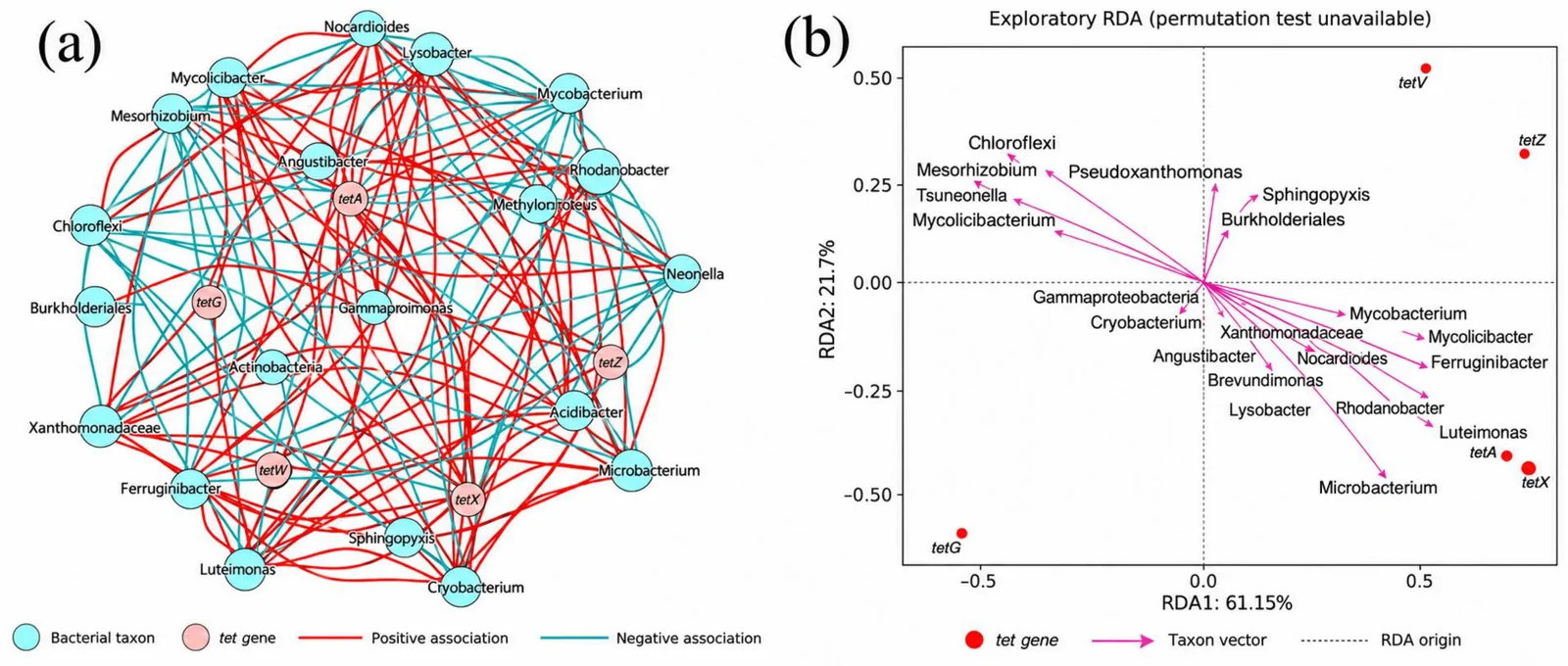
Exploratory associations between tetracycline resistance genes (TRGs) and microbial taxa: (**a**) Spearman co-occurrence network (red, positive; blue, negative) and (**b**) redundancy analysis. The RDA permutation test must be completed before inferential interpretation.

The correlation network between the detected TRGs and selected bacterial taxa in the TC–Cu co-contaminated soils is presented in Figure 9a. Red and cyan edges represent positive and negative associations, respectively. The dense connectivity of *tetA*, *tetX*, *tetZ*, and other TRGs with taxa such as *Rhodanobacter*, *Mycobacterium*, and *Microbacterium* indicates coordinated variation in their abundances across the treatments. The co-occurrence of *Rhodanobacter* with several TRGs may reflect a shared response to TC–Cu selection pressure or indirect ecological interactions rather than direct carriage of *tetA* or other resistance genes [7]. Similarly, *Lysobacter* was connected to the network through both positive and negative edges; therefore, the network does not support the conclusion that this genus consistently reduced TRG abundance by inhibiting antibiotic-resistant bacteria. Differences in the connectivity of Chloroflexi, Xanthomonadaceae, and other taxa indicate variation in their statistical associations with TRGs, but edge number and direction cannot establish their direct involvement in horizontal gene transfer (HGT) [25]. An exploratory redundancy analysis (RDA) of the relationships between TRGs and bacterial taxa is shown in Figure 9b. RDA1 and RDA2 explained 61.15% and 21.70% of the constrained variation, respectively, accounting for a cumulative 82.85%. *TetV* and *tetZ* were positioned in the upper-right quadrant and were oriented more closely toward taxa such as Burkholderiales and *Sphingopyxis*. By contrast, *tetA*, *tetX*, and *tet36* occurred in the lower-right quadrant, broadly corresponding to the directions of *Microbacterium*, *Luteimonas*, and *Rhodanobacter* [35]. *TetG* was separated from the other TRGs in the lower-left quadrant, suggesting that its distribution differed from those of the remaining resistance genes. Gammaproteobacteria was located close to the RDA origin and had a comparatively short vector, indicating a relatively weak directional relationship with the ordination axes. Integration of the TRG, microbial-community, and remediation results suggests that CP amendment was associated with enhanced TC dissipation, reduced bioavailable Cu, lower total TRG abundance, and substantial restructuring of the bacterial community. CP amendment reduced the overall contaminant burden and altered microbial-community composition, which collectively coincided with a decrease in total TRG abundance.

## 4. Conclusions

Within the 49-d microcosms, *C. pyrenoidosa* amendment was associated with increased dissipation of parent TC, a 35.6–50.6% decrease in extractable Cu, partial recovery of soil enzyme activities, and shifts in bacterial community and TRG profiles. The highest TC dissipation was reported in TC30Cu100CP, whereas the largest decrease in extractable Cu occurred in TC100Cu100CP. The results support the potential use of microalgal amendment to reduce pollutant bioavailability in TC-Cu co-contaminated soil. This 49-day microcosm study demonstrated that TC–Cu co-contamination substantially disturbed soil ecological quality, as reflected by decreases in soil pH and organic matter, elevated electrical conductivity, inhibition of dehydrogenase, catalase, and nitrate reductase activities, and pronounced shifts in bacterial community composition. Cu generally exerted stronger biological inhibition than TC, while co-exposure increased the total abundance of tetracycline resistance genes (TRGs), suggesting enhanced co-selection pressure under combined contamination. Amendment with CP effectively attenuated these adverse effects. TC dissipation increased to 49.9–73.5%, with the highest value observed in TC30Cu100CP, whereas bioavailable Cu was reduced by 35.6–50.6%, with the greatest reduction occurring in TC100Cu100CP. CP amendment also partially restored soil physicochemical properties and enzyme activities and substantially reshaped the bacterial community. Total TRG abundance decreased by approximately 36–57% relative to the corresponding unamended treatments, with the greatest absolute reduction of approximately 4.8 × 10^8^ copies g^−1^ DW observed in TC100Cu500CP. Although *tetX* remained the predominant detected TRG, its abundance was markedly reduced following CP amendment. The remediation effects of CP may have resulted from the combined contributions of contaminant sorption and immobilization, reduced contaminant bioavailability, improved soil microenvironmental conditions, and restructuring of microbial communities. Overall, *C. pyrenoidosa* shows considerable potential as a biological amendment for mitigating antibiotic–metal co-contamination and reducing the associated soil resistance burden. Further field-scale studies and direct analyses of EPS-mediated immobilization, TRG host assignment, mobile genetic elements, and horizontal gene transfer are required to validate the proposed mechanisms and assess the long-term ecological safety of this remediation strategy.

## Figures and Tables

**Table 1:** Factorial treatment design used to assess *Chlorella pyrenoidosa* supplementation in tetracycline–copper co-contaminated soil.

Groups	TC (mg·kg^−1^)	Cu (mg·kg^−1^)	*Chlorella pyrenoidosa* (mL)
CK	0	0	0
CKCP	0	0	6.96
TC30	30	0	0
TC30CP	30	0	6.96
TC100	100	0	0
TC100CP	100	0	6.96
Cu100	0	100	0
Cu100CP	0	100	6.96
Cu500	0	500	0
Cu500CP	0	500	6.96
TC30Cu100	30	100	0
TC30Cu100CP	30	100	6.96
TC30Cu500	30	500	0
TC30Cu500CP	30	500	6.96
TC100Cu100	100	100	0
TC100Cu100CP	100	100	6.96
TC100Cu500	100	500	0
TC100Cu500CP	100	500	6.96

## Data Availability

The raw data supporting the conclusions of this article will be made available by the authors on request.

## Abbreviations

The following abbreviations are used in this manuscript:

ARGs	Antibiotic Resistance Genes
ANOVA	Analysis of Variance
CK	Control (uncontaminated soil)
CP	*Chlorella pyrenoidosa*
Cu	Copper
DTPA	Diethylenetriaminepentaacetic acid
EC	Electrical Conductivity
EPS	Extracellular Polymeric Substances
HGT	Horizontal Gene Transfer
HPLC	High-Performance Liquid Chromatography
ICP-MS	Inductively Coupled Plasma Mass Spectrometry
LDA	Linear Discriminant Analysis
LEfSe	Linear Discriminant Analysis Effect Size
NR	Nitrate Reductase
ORF	Open Reading Frame
RDA	Redundancy Analysis
S-CAT	Soil Catalase
SOM	Soil Organic Matter
SPE	Solid-Phase Extraction
TC	Tetracycline
TPM	Transcripts Per Million
TRGs	Tetracycline Resistance Genes
TTC	Triphenyltetrazolium Chloride
